# BDNF Deficiency Preserves Shoal Structure but Selectively Modulates Horizontal Exploration in an Adult BDNF^−/−^ Zebrafish Line

**DOI:** 10.3390/ijms27125464

**Published:** 2026-06-17

**Authors:** Amalys Sofia Sanchez Garcia, Flavia Frabetti, Giulia Brighi, Gabriella Tedeschi, Arianna Racca, Enrico Alleva, Mattia Toni

**Affiliations:** 1 Department of Biology and Biotechnologies “Charles Darwin”, Sapienza University, 00185 Rome, Italy; sanchezgarcia.2065678@studenti.uniroma1.it; 2 Department of Medical and Surgical Sciences (DIMEC), University of Bologna, 40126 Bologna, Italy; flavia.frabetti@unibo.it (F.F.); giulia.brighi5@unibo.it (G.B.); 3 Department of Veterinary Medicine and Animal Science (DIVAS), Università degli Studi di Milano, Via dell’Università 6, 26900 Lodi, Italy; gabriella.tedeschi@unimi.it; 4 CRC “Innovation for Well-Being and Environment” (I-WE), Università degli Studi di Milano, 20126 Milan, Italy; 5 Centre for Behavioural Sciences and Mental Health, Istituto Superiore di Sanità, Viale Regina Elena 299, 00161 Rome, Italy; arianna.racca@iss.it (A.R.); enrico.alleva@guest.iss.it (E.A.)

**Keywords:** BDNF, zebrafish, *Danio rerio*, behaviour, shoaling, environment

## Abstract

Brain-derived neurotrophic factor (BDNF) is a key regulator of neural development, plasticity, and behaviour. Recent work has enabled the generation of a viable adult *bdnf*^−/−^ zebrafish line, which provides a unique opportunity to investigate how complete loss of *bdnf* affects social behaviour. Here, we examined three-dimensional shoaling behaviour in adult male and female AB wild-type and *bdnf*^−/−^ knock-out zebrafish to determine whether the extensive molecular and behavioural alterations previously observed in individual-based assays extend to collective contexts. The *bdnf*^−/−^ shoals showed no differences in group structure, as inter-fish distance, shoal volume, shoal area, distance to the centroid, and homogeneity index were comparable to wild-type groups. Vertical spatial distribution was also largely preserved, although *bdnf*^−/−^ fish shifted toward the upper regions of the tank earlier during the trial. By contrast, horizontal distribution revealed a clear genotype effect: *bdnf*^−/−^ shoals spent more time in peripheral regions and displayed a pronounced early peak in peripheral occupancy. These findings indicate that *bdnf* loss does not impair shoal formation or cohesion but selectively modulates specific components of spatial exploration. The results also highlight a dissociation between the vertical and horizontal axes of behaviour, as well as between individual- and group-based phenotypes, underscoring the importance of social context in shaping the behavioural consequences of BDNF deficiency.

## 1. Introduction

Brain-derived neurotrophic factor (BDNF) is a central regulator of neuronal development, synaptic plasticity, and circuit refinement across vertebrates [1,2,3]. Beyond its well-established developmental functions, *bdnf* continues to modulate sensory processing, emotional regulation, and behavioural output in the adult brain [1]. Altered BDNF signalling has therefore been implicated in a wide range of neuropsychiatric and neurodevelopmental conditions [4,5], highlighting the importance of understanding how this neurotrophin shapes neural function at both molecular and system levels.

The zebrafish (*Danio rerio*) has emerged as a powerful vertebrate model for dissecting the molecular- and circuit-level mechanisms underlying behaviour. A broad repertoire of validated behavioural paradigms—including assays of anxiety, exploration, social interaction, and cognition—combined with high-throughput omics technologies (e.g., transcriptomics, proteomics, and miRNA profiling) enables a multi-level characterisation of neurobiological phenotypes [6,7,8,9,10,11]. Zebrafish also display rich social behaviours [12], such as shoaling [13,14,15], a form of collective organisation that depends on the integration of sensory cues, internal state, and coordinated motor output [14,16,17]. Because shoaling is sensitive to perturbations in neural function, it provides an ethologically relevant readout for assessing the behavioural consequences of molecular and cellular alterations [15].

Our group recently generated and characterised a viable adult *bdnf*^−/−^ zebrafish line [18,19]. Multi-omics analyses revealed widespread dysregulation of transcripts and proteins involved in synaptic transmission, cytoskeletal organisation, vesicle trafficking, energy metabolism, and oxidative stress regulation, indicating large-scale remodelling of neural architecture in the absence of *bdnf* [18]. Behavioural studies across multiple assays, including the Y-maze test (YMT), novel tank diving test (NTT) [20], light–dark preference test (LDT) [21,22], open field test [OFT], mirror biting test (MBT) [23], social interaction test (SIT), and social preference test (SPT), demonstrated marked alterations in locomotion, anxiety-like responses, stress reactivity, circadian rhythmicity, and cognitive performance in both larvae and adults [18,19]. Together, these findings suggest that BDNF deficiency profoundly affects neural systems supporting sensory integration, arousal, and behavioural regulation.

Despite the strong social nature of zebrafish and the central role of social interaction in their ecology, the impact of complete *bdnf* loss on collective social behaviour has not been systematically investigated. Whether the extensive molecular and behavioural alterations previously described in isolated individuals extend to group-level dynamics remains unknown. The present study addresses this gap by quantifying three-dimensional shoaling behaviour in adult wild-type (WT) and *bdnf*^−/−^ knock-out (KO) zebrafish, providing a functional and behavioural extension of the molecular characterisation previously reported for this line [18,19].

## 2. Results and Discussion

**Figure 1:**
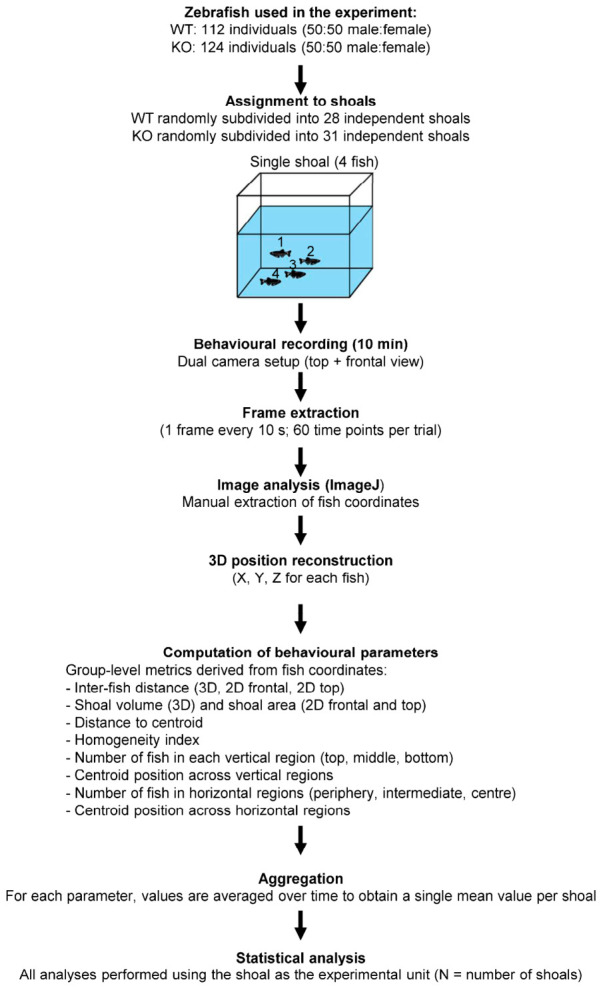
**Schematic representation of the experimental design and data analysis workflow.** Adult zebrafish were tested in shoals of four individuals (single shoal = experimental unit) in a rectangular tank. Behaviour was recorded during a 10 min trial using a dual-camera setup (top and frontal views). Video recordings were processed by extracting frames at 10 s intervals across the entire duration of the test (60 time points per shoal). Fish positions were obtained from each frame using image analysis (ImageJ), allowing reconstruction of three-dimensional spatial coordinates (X, Y, Z) for each individual. Group-level behavioural parameters were then computed based on spatial relationships among the four fish, including inter-fish distance, shoal volume and area, distance to the centroid, homogeneity index, and spatial distribution across vertical (top, middle, bottom) and horizontal (centre, intermediate, periphery) regions. For each parameter, values were averaged over time to obtain a single mean value per shoal. Statistical analyses were performed using the shoal as the unit of replication (N = number of independent shoals).

Spatio-temporal shoaling behaviour in adult zebrafish was examined by comparing KO and WT (control) groups. The analysis focused on two complementary aspects of collective behaviour: shoal organisation and spatial positioning within the experimental arena (Figure 1).

**Figure 2:**
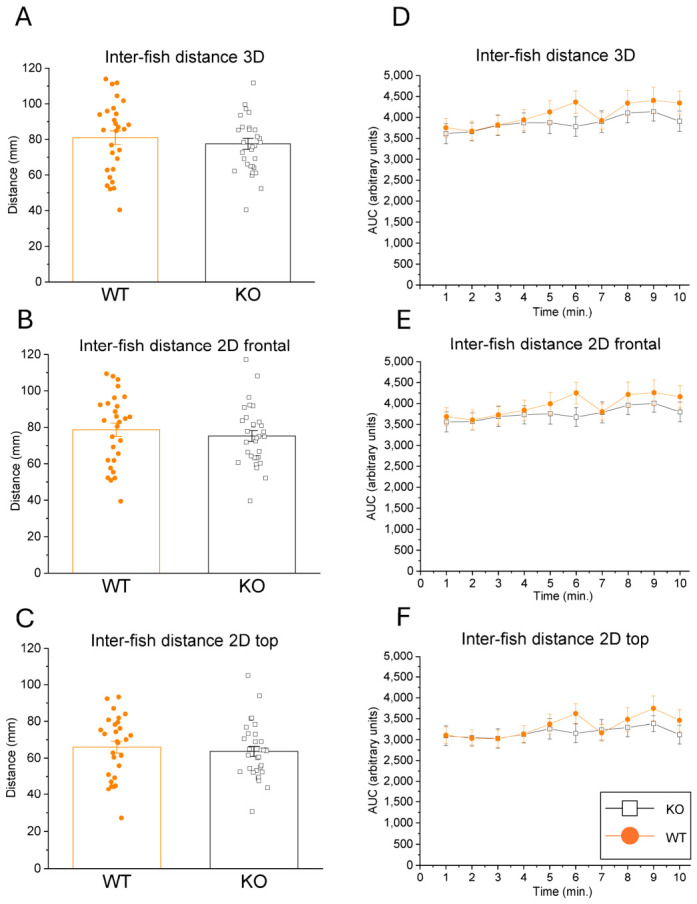
**Effect of *bdnf* loss and time on inter-fish distance.** Inter-fish distance in 3D space (**A**), 2D frontal plane (**B**), and 2D top view (**C**) for WT and KO shoals. Data are presented as scatter plots, where each point represents one independent shoal (WT, n = 28; KO, n = 31), with mean ± S.E.M. indicated. For each shoal, values correspond to the mean inter-individual distance averaged across all sampled time points of the trial. Temporal modulation across the 10 min trial is shown as AUC values for 3D distance (**D**), frontal plane distance (**E**), and top plane distance (**F**). Data are presented as mean ± S.E.M. Statistical details are reported in Table 1 and Tables S1–S3. Statistical significance was set at *p* ≤ 0.05. Significance symbols, as defined in Section 3.3 (Statistical Analysis), appear only when the corresponding effect is significant and may therefore not be present in all panels. Colour/symbol coding: KO = black squares; WT = orange circles.

**Figure 3:**
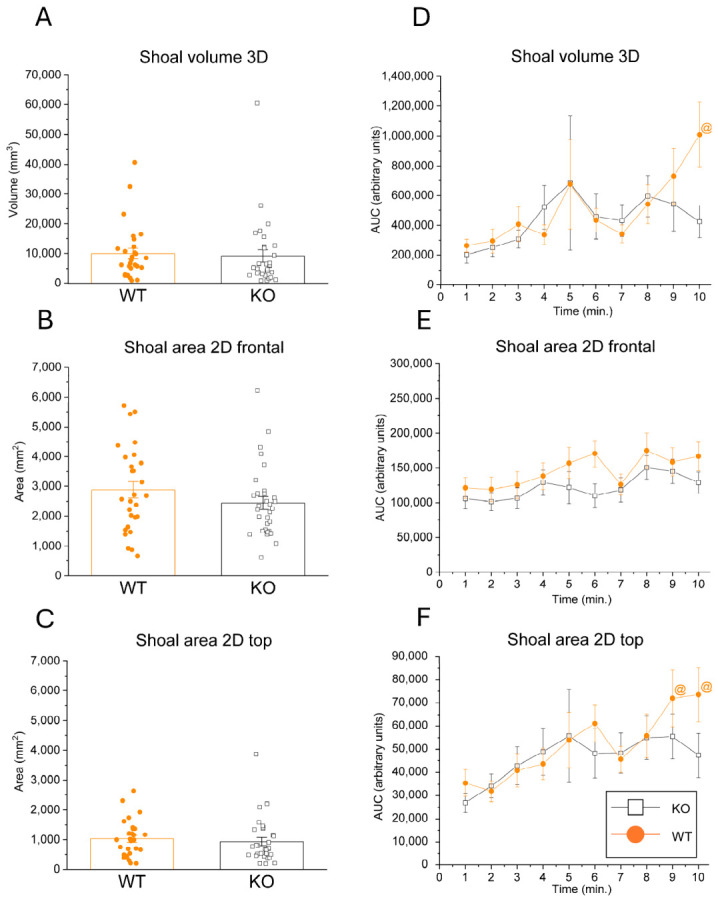
**Effect of *bdnf* loss and time on shoal dimensions.** Shoal volume in 3D space (**A**), shoal area in the 2D frontal plane (**B**), and shoal area in the 2D top view (**C**) for WT and KO shoals. Each point represents one independent shoal (WT, n = 28; KO, n = 31), and values correspond to the mean measurements averaged across all sampled time points of the trial. Data are presented as mean ± S.E.M. Temporal modulation expressed as AUC values for the same parameters is shown in panels (**D**–**F**). Data are presented as mean ± S.E.M. Statistical details are reported in Table 1 and Tables S1–S3. In panels (**D**,**F**) @ indicate significant Time effects relative to minute 1 in WT fish.

**Figure 4:**
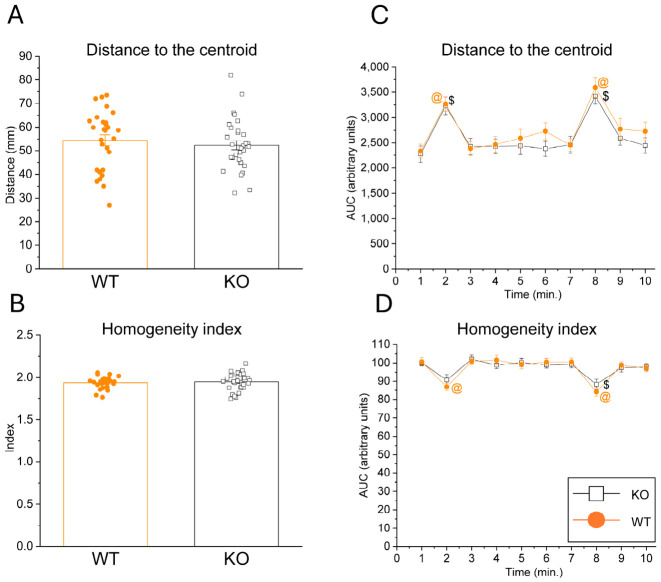
**Effect of *bdnf* loss and time on shoal cohesion.** Mean distance to the centroid (**A**) and homogeneity index (**B**) for WT and KO shoals. Each point represents one independent shoal (WT, n = 28; KO, n = 31), and values correspond to measurements averaged across all sampled time points of the trial, reflecting overall group cohesion. Temporal modulation expressed as AUC values for the parameters is shown in panels (**C**,**D**). Data are presented as mean ± S.E.M. Statistical details are reported in Table 1 and Tables S1–S3. Significance symbols as in Figure 2. In panels (**C**,**D**), $ and @ indicate significant Time effects relative to minute 1 in KO and WT fish, respectively.

Shoal organisation was characterised by assessing both group size-related features and internal cohesion. Group size was quantified through multiple parameters, including mean inter-individual distance calculated in three-dimensional space as well as along the vertical and horizontal axes (Figure 2), overall shoal volume, and shoal area measured separately on the vertical and horizontal planes (Figure 3). Moreover, shoal cohesion was evaluated using distance to the group centroid and a homogeneity index (Figure 4), which together provide information on the degree of compactness and uniformity within the shoal [24].

**Figure 5:**
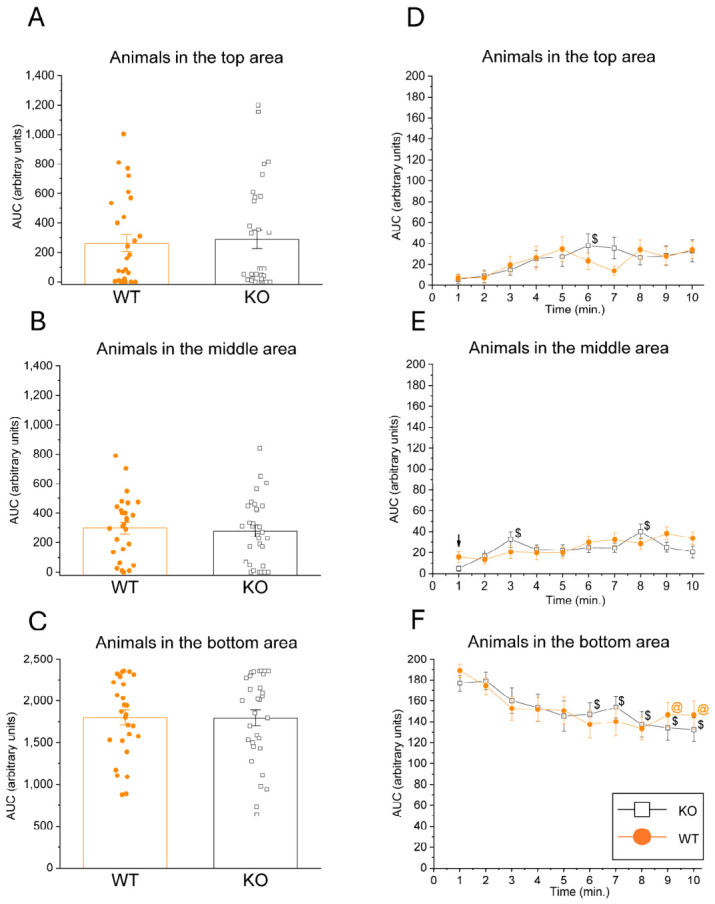
**Effect of *bdnf* loss and time on vertical distribution based on the position of shoal members**. Mean number of zebrafish in the top (**A**), middle (**B**), and bottom (**C**) zones for WT and KO shoals. Values represent the mean number of individuals per zone, calculated for each sampled time point (every 10 s) and averaged across the entire observation period for each shoal (mean occupancy). Each point corresponds to one independent shoal (WT, n = 28; KO, n = 31). Temporal modulation expressed as AUC values for the same parameters is shown in panels (**D**–**F**). Data are presented as mean ± S.E.M. Statistical details are reported in Table 1 and Tables S1–S3. $ and @ indicate significant Time effects relative to minute 1 in KO and WT fish, respectively. Black arrow mark time points showing a significant Genotype × Time interaction.

**Figure 6:**
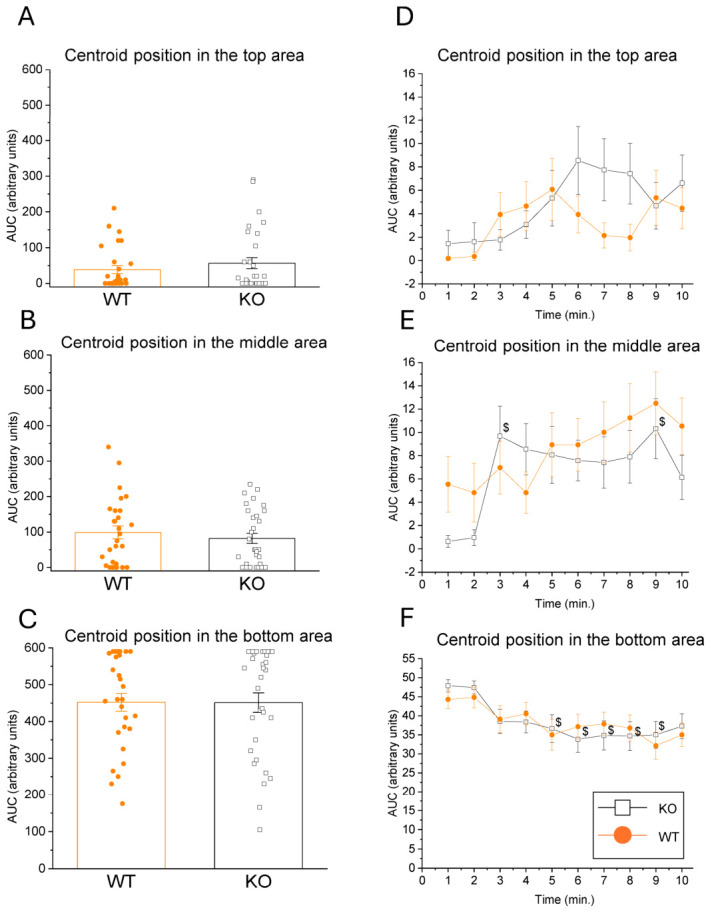
**Effect of *bdnf* loss and time on vertical distribution based on centroid position.** Mean centroid position in the top (**A**), middle (**B**), and bottom (**C**) zones for WT and KO shoals. Each point represents one independent shoal (WT, n = 28; KO, n = 31), with values averaged across all sampled time points of the trial. Temporal modulation expressed as AUC values for the same parameters is shown in panels (**D**–**F**). Data are presented as mean ± S.E.M. Statistical details are reported in Table 1 and Tables S1–S3. In panels (**E**,**F**) $ indicate significant Time effects relative to minute 1 in KO fish.

**Figure 7:**
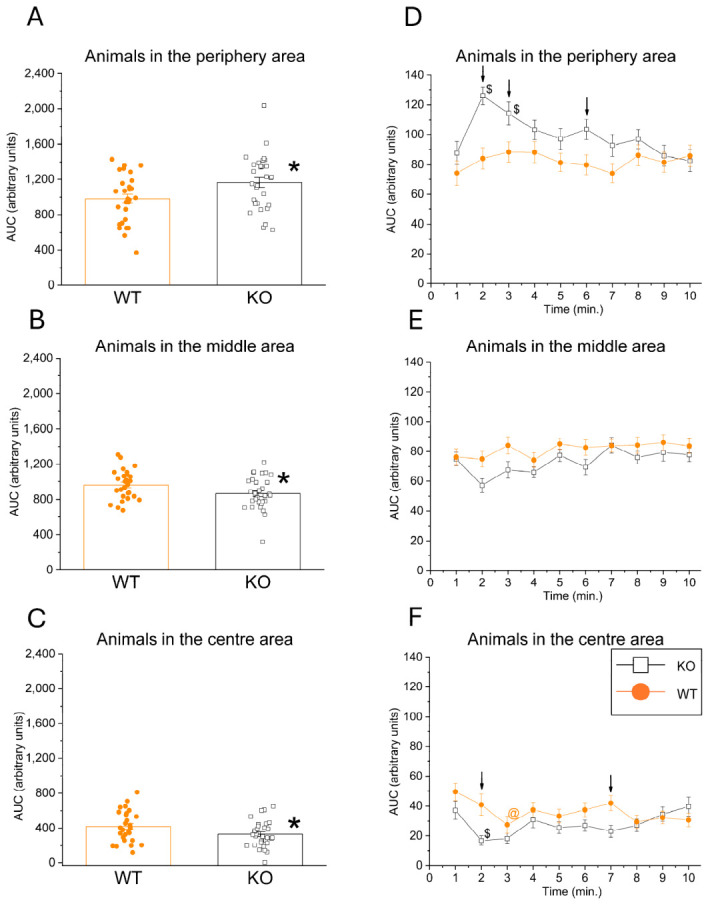
**Effect of *bdnf* loss and time on horizontal distribution based on the position of shoal members**. Mean number of zebrafish in the periphery (**A**), middle (**B**), and centre (**C**) zones for WT and KO shoals. Values represent the mean number of individuals per zone, calculated at each sampled time point (every 10 s) and averaged across the full observation period for each shoal (mean occupancy). Each point represents one independent shoal (WT, n = 28; KO, n = 31). Data are presented as mean ± S.E.M. Temporal modulation expressed as AUC values for the same parameters is shown in panels (**D**–**F**). Statistical details are reported in Table 1 and Tables S1–S3. * denotes significant Genotype effects (KO vs. WT). $ and @ indicate significant Time effects relative to minute 1 in KO and WT fish, respectively. Black arrows mark time points showing a significant Genotype × Time interaction.

**Figure 8:**
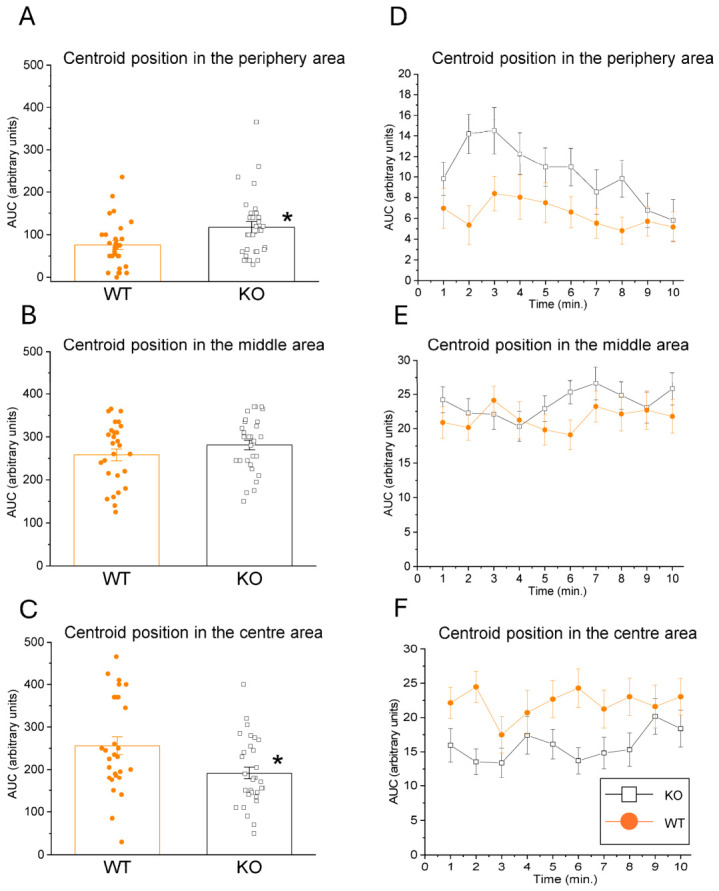
**Effect of *bdnf* loss and time on horizontal distribution based on centroid position.** Mean centroid position in the periphery (**A**), middle (**B**), and centre (**C**) zones for WT and KO shoals. Each point represents one independent shoal (WT, n = 28; KO, n = 31), with values averaged across all sampled time points of the trial. Data are presented as mean ± S.E.M. Temporal modulation expressed as AUC values for the same parameters is shown in panels (**D**–**F**). Data are presented as mean ± S.E.M. Statistical details are reported in Table 1 and Tables S1–S3. * denotes significant Genotype effects (KO vs. WT).

Shoal positioning was analysed by examining the spatial distribution of fish across the vertical (Figure 5 and Figure 6) and horizontal (Figure 7 and Figure 8) dimensions of the tank, allowing detection of genotype-dependent preferences in space use. To determine whether the absence of *bdnf* influenced shoaling behaviour, statistical comparisons between KO and WT groups were performed using one-way ANOVA, with genotype as the independent factor (Table 1).

The temporal dynamics of shoaling behaviour were then investigated using one-way repeated-measures ANOVA to assess the contribution of time (i.e., minute-by-minute changes during the ten-minute observation period) to each parameter. To summarise behavioural trends across the entire trial, analyses were conducted on area under the curve (AUC) values. Where appropriate, Bonferroni’s post hoc correction was used to identify the specific time points at which significant within-genotype changes occurred.

Finally, to determine whether WT and KO shoals differed in their temporal trajectories, all parameters were also analysed using a two-way repeated-measures ANOVA (Genotype × Time), followed by Bonferroni-corrected contrasts to identify the specific minutes at which the two genotypes diverged.

This multi-level analytical approach enabled a detailed evaluation of how shoal structure and spatial positioning evolved over time in WT and KO zebrafish, providing insight into the role of BDNF in the regulation of collective behaviour.

### 2.1. Shoal Structure and Cohesion

Inter-fish distance did not differ between WT and KO shoals in any spatial dimension (3D, frontal, or top view; Figure 2A–C; Table 1). Shoal volume and 2D shoal area were likewise comparable between genotypes (Figure 3A–C; Table 1). These findings indicate that *bdnf* deletion does not alter shoal structure or inter-individual spacing, suggesting that the absence of *bdnf* does not reduce interest in conspecifics. This interpretation aligns with previous single subject assays—including the social preference test [18] and the mirror based social interaction test [25]—which similarly failed to detect reduced conspecific directed interest in KO individuals.

Because anxiogenic compounds typically reduce inter-fish distance and shoal area, whereas anxiolytics increase them [24], the absence of genotype effects suggests that the observed behavioural pattern is not consistent with typical anxiety-like modulation under standard conditions. This contrasts with the bold behavioural profile previously observed in KO individuals in single-fish assays [18], suggesting that such traits do not necessarily manifest when fish interact within a social group.

WT shoals showed significant temporal modulation in several structural parameters, including inter-fish distance (3D: F_(9,243)_ = 2.38, *p* = 0.0136; 2D frontal: F_(9,243)_ = 1.95, *p* = 0.0461; 2D top: F_(9,243)_ = 2.15, *p* = 0.0260), shoal volume (F_(2.97,80.33)_ = 3.50, *p* = 0.0195) and shoal area (frontal: F_(9,243)_ = 2.26, *p* = 0.0189; top: F_(5.06,136.48)_ = 4.42, *p* = 0.0009) (Figure 2D–F; Figure 3D–F; Table 1).

However, Bonferroni-corrected post hoc comparisons did not reveal robust pairwise differences between specific minutes, indicating that these temporal changes were relatively subtle. KO shoals did not exhibit comparable time dependent variation, resulting in a more stable structural profile across the trial. Overall, temporal modulation in shoal structure was more pronounced in WT groups.

Distance to the centroid and the homogeneity index were also comparable between genotypes (Figure 4A,B; Table 1). Both WT and KO shoals exhibited similar temporal modulation in centroid distance (WT: F_(9,243)_ = 11.24, *p* < 0.0001; KO: F_(9,270)_ = 8.47, *p* < 0.0001) and homogeneity (WT: F_(9,243)_ = 8.44, *p* < 0.0001; KO: F_(6.10,183.13)_ = 4.22, *p* = 0.0005) (Figure 4C,D; Figure S1; Table 1). These patterns were characterised by two transient peaks in centroid distance at minutes 2 and 8, accompanied by corresponding reductions in homogeneity. Full statistical details are provided in Table S1.

A finer-grained temporal analysis (Figure S1) revealed that these peaks were not uniformly distributed across the entire minute but were primarily driven by rapid increases occurring within the first ~10 s of minutes 2 and 8, followed by a sustained plateau lasting most of the interval and a gradual decline towards baseline levels in the final seconds.

Importantly, no external disturbances occurred during these time points, as behavioural recordings were conducted under controlled conditions with no experimenter present in the room during data acquisition.

These brief reductions in group compactness were shared by both genotypes and do not resemble the sustained decreases in centroid distance typically associated with anxiogenic states [24] but likely reflect early-phase exploratory adjustments. Taken together, BDNF deficiency does not appear to affect shoal cohesion or internal dispersion.

The two-way repeated measures ANOVA did not reveal Genotype × Time interactions for any structural or cohesion related parameter (Table 1). Despite the presence of temporal modulation in WT shoals, the overall temporal trajectories of WT and KO groups were comparable. The absence of interaction effects suggests that the reduced temporal modulation in KO shoals reflects lower within-group variability rather than a genotype-specific limitation in temporal flexibility.

### 2.2. Spatial Positioning

#### 2.2.1. Vertical Spatial Distribution

Vertical positioning was assessed using both the number of individuals (Figure 5) and the centroid location (Figure 6) within the top, middle, and bottom zones. No genotype effect emerged for any of these parameters (Figure 5A–C and Figure 6A–C; Table 1). KO and WT shoals did not differ in the proportion of fish occupying the top, middle, or bottom regions, indicating that *bdnf* loss does not alter the group’s vertical preference profile. Given that anxiogenic compounds typically reduce top-zone exploration and anxiolytics increase it [24], the absence of genotype differences suggests that KO shoals do not display a more anxiogenic or anxiolytic like vertical pattern than WT groups.

This result contrasts with previous findings in single-subject assays, where KO individuals showed increased exploration of the upper-tank region relative to WT fish [18]. The discrepancy highlights that vertical behaviour expressed by isolated individuals does not necessarily translate into altered vertical positioning at the shoal level.

Both genotypes exhibited clear time-dependent modulation in vertical distribution. Significant effects were observed in the top zone (KO: F_(3.61,108.16)_ = 2.62, *p* = 0.0445), middle zone (WT: F_(4.99,134.79)_ = 2.99, *p* = 0.0136; KO: F_(4.57,136.97)_ = 4.45, *p* = 0.0013), and bottom zone (WT: F_(3.52,95.12)_ = 3.39, *p* = 0.0158; KO: F_(3.94,118.26)_ = 3.83, *p* = 0.0060). Centroid position also showed significant temporal modulation, particularly in KO shoals (middle: F_(5.03,151.01)_ = 3.61, *p* = 0.0041; bottom: F_(3.90,116.92)_ = 4.46, *p* = 0.0024) (Figure 5D–F and Figure 6D–F; Table 1). Full statistical details, including effect sizes and power, are reported in Table S1.

WT and KO shoals progressively reduced bottom-zone occupancy while increasing time spent in the middle and top zones, consistent with the typical habituation trajectory in zebrafish, which initially remain in deeper regions before gradually exploring upper areas [8,20,26].

A finer-grained inspection revealed subtle temporal differences between genotypes. KO shoals shifted upward earlier than WT shoals, with Bonferroni-corrected post hoc tests indicating significant increases in middle-zone occupancy as early as minute 3, followed by reduced bottom-zone occupancy and a later rise in top-zone occupancy (Table 1). Although the final vertical distribution was comparable between genotypes, KO shoals adjusted their depth preference more rapidly, suggesting enhanced initial reactivity or faster habituation—consistent with the bolder phenotype previously described in individual-based assays [18].

The two-way repeated-measures ANOVA confirmed a transient Genotype × Time interaction for middle-zone occupancy (F_(5.72,325.86)_ = 2.19, *p* = 0.0470) (Table 1). However, this divergence was short-lived, and the overall vertical habituation pattern remained largely similar between genotypes. These findings indicate that *bdnf* loss does not substantially alter the shoal’s vertical distribution, although KO fish show a slightly accelerated early-phase adjustment without modifying the global vertical exploration strategy.

#### 2.2.2. Horizontal Spatial Distribution

Horizontal positioning was the only domain showing a clear and robust genotype effect. Both the distribution of individuals (Figure 7) and centroid location (Figure 8) revealed that KO shoals spent more time in the periphery (number of animals: F_(1,57)_ = 5.79, *p* = 0.0194, η^2^ = 0.092; centroid position F_(1,57)_ = 5.57, *p* = 0.0218, η^2^ = 0.089) and less time in the centre (number of animals: F_(1,57)_ = 4.43, *p* = 0.0398, η^2^ = 0.072; centroid position: F_(1,57)_ = 6.67, *p* = 0.0124, η^2^ = 0.105) compared with WT shoals. A smaller but significant effect was also observed in the intermediate region (F_(1,57)_ = 4.42, *p* = 0.0399, η^2^ = 0.072), indicating an overall shift toward the outermost region of the tank (Figure 7A–C and Figure 8A–C; Table 1). These effect sizes fall within the moderate range, (η^2^ ≈ 0.07–0.10), indicating that genotype accounts for a consistent and biologically meaningful proportion of variance in horizontal spatial distribution. Importantly, these effects were observed across both individual-based measures and centroid-based metrics, supporting the robustness of the genotype-dependent shift. Despite partial overlap between individual observations, the data indicate a systematic redistribution of spatial occupancy rather than a discrete separation between groups.

Temporal analysis showed that WT shoals maintained a stable horizontal configuration throughout the trial, with no significant modulation in periphery or intermediate occupancy. A modest effect was detected in the centre (F_(9,243)_ = 2.27, *p* = 0.0188) (Figure 7D–F and Figure 8D–F; Table 1). By contrast, KO shoal members exhibited pronounced time-dependent changes across all regions, including the periphery (F_(5.64,169.31)_ = 6.14, *p* < 0.0001), intermediate zone (F_(9,270)_ = 3.75, *p* = 0.0002), and centre (F_(5.07,151.95)_ = 3.33, *p* = 0.0068). Centroid position in the periphery also showed significant temporal modulation (F_(5.70,171.09)_ = 3.25, *p* = 0.0055). KO fish displayed a sharp early peak in peripheral occupancy at minutes 2–3 (Bonferroni-corrected), accompanied by reduced occupancy in the intermediate and centre regions, followed by a gradual decline in peripheral use later in the trial. This transient early exploitation of the periphery was not observed in WT shoals. Full statistical details, including effect sizes and power, are reported in Table S1.

These dynamics suggest that KO fish respond to the novel environment with an initial enhancement of peripheral exploration, whereas WT shoals maintain a more stable horizontal distribution. This pattern differs from previous open field tests in single adult subjects, where no genotype differences in thigmotaxis were detected [25], highlighting again that group-level behaviour does not always mirror individual-level responses.

Although anxiolytic and anxiogenic treatments typically modulate vertical rather than horizontal distribution, it has been reported that anxiolytics can increase peripheral occupancy over time, whereas anxiogenic compounds reduce it [24]. The early peripheral peak observed in KO shoals resembles the temporal profile associated with reduced anxiety or increased boldness, consistent with previous findings in individual based assays [18].

The two-way repeated-measures ANOVA revealed a significant Genotype × Time interaction for horizontal distribution in both the periphery (F_(6.37,363.07)_ = 2.51, *p* = 0.0190) and the centre (F_(6.85,390.63)_ = 2.45, *p* = 0.0188) (Table 1). Full statistical details, including effect sizes are reported in Table S1.

KO shoals showed a marked early increase in peripheral occupancy at minutes 2–3, whereas WT shoals remained stable across the trial. These interaction effects indicate that horizontal exploration is the only spatial domain in which *bdnf* loss alters both the overall spatial preference and its temporal dynamics, producing a transient early-phase divergence between genotypes.

### 2.3. Broader Interpretative Considerations: Two Key Discrepancies

The present study shows that *bdnf* loss does not disrupt shoal structure, cohesion, or vertical distribution but selectively alters the temporal dynamics of horizontal exploration. KO shoals remain cohesive and vertically indistinguishable from WT groups, yet they display an early, transient increase in peripheral occupancy in the horizontal plane. These findings reveal a nuanced behavioural phenotype in which the core features of social organisation are preserved, while specific aspects of exploratory strategy are modified.

Beyond these specific outcomes, the behavioural analysis highlights two broader discrepancies that warrant deeper consideration. First, *bdnf* loss selectively affected horizontal—but not vertical—exploration, despite both axes contributing to the overall spatial dynamics of the shoal. Second, the behavioural phenotype of BDNF-deficient fish differed markedly depending on whether animals were tested individually or within a group: KO zebrafish displayed pronounced alterations in multiple individual-based assays, yet these differences were substantially attenuated in the shoaling context.

To contextualise these discrepancies, it is useful to consider the broader molecular landscape previously characterised in this *bdnf*^−/−^ line. Larval transcriptomics revealed early dysregulation of neurodevelopmental, synaptic, circadian, and neurotrophin-related pathways [19], while adult whole-brain proteomics demonstrated reduced abundance of synaptic, cytoskeletal, vesicle trafficking, and neurotransmission-related proteins, together with alterations in energy metabolism and oxidative stress regulation [18]. These molecular signatures indicate that BDNF deficiency induces widespread remodelling of neural systems involved in sensory integration, arousal regulation, and behavioural control.

**Figure 9:**
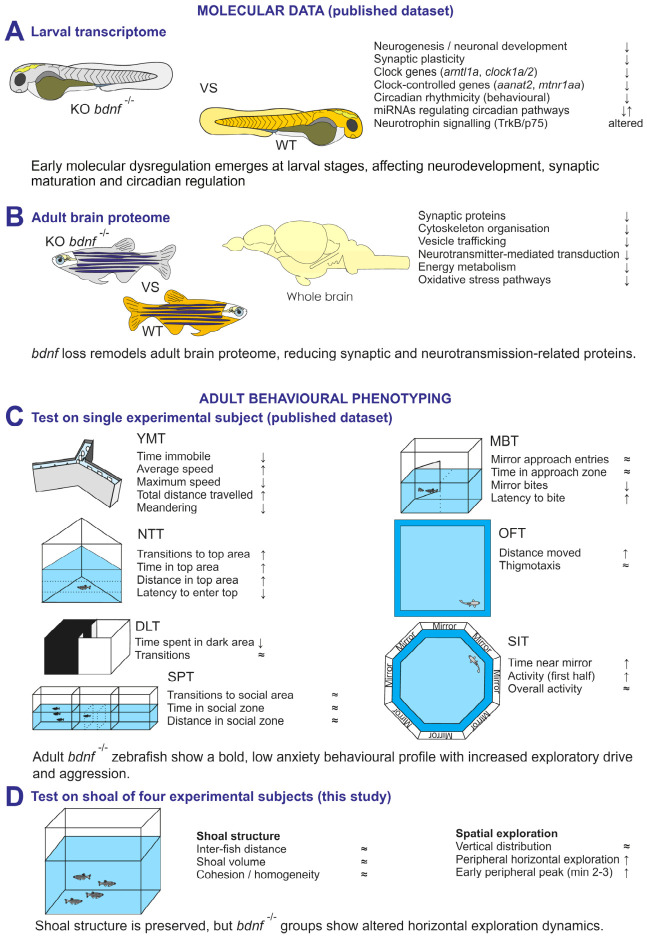
**Summary of molecular alterations and behavioural phenotypes associated with *bdnf* loss in zebrafish.** (**A**) Larval transcriptome. Transcriptomic analyses (published dataset [19]) reveal early dysregulation of neurodevelopmental, synaptic, circadian, neurotrophin signalling, and metabolic pathways in *bdnf*^−/−^ zebrafish. (**B**) Adult brain proteome. Proteomic analysis of adult brain (published dataset [18]) shows reduced abundance of synaptic, cytoskeletal, vesicle trafficking, neurotransmission-related, and metabolic proteins, together with increased oxidative stress pathways, indicating extensive remodelling of neural architecture. (**C**) Adult behavioural phenotyping in single fish. Behavioural assays (published datasets [18,25]) include the Y-maze test (YMT), novel tank diving test (NTT), light–dark preference test (LDT), open field test (OFT), mirror biting test (MBT), social interaction test (SIT), and social preference test (SPT). These analyses demonstrate increased locomotor activity, enhanced exploration of upper tank regions, reduced anxiety-like behaviour in the light–dark test, increased sociability in mirror-based assays, and heightened aggression in mirror biting tests, while social preference for live conspecifics remains unchanged. Open field testing confirms elevated activity with preserved thigmotaxis. (**D**) Shoaling behaviour (this study). Group-based analysis shows that core group shoal structure is preserved in *bdnf*^−/−^ fish, while horizontal spatial exploration is selectively altered, with increased peripheral occupancy and the presence of an early peripheral peak. Together, these data indicate that BDNF deficiency induces widespread molecular alterations during development that translate into distinct behavioural phenotypes at both individual and group levels.

Figure 9 integrates these molecular alterations with the behavioural phenotypes observed in adults across individual based and shoaling assays. This synthesis highlights how *bdnf* loss produces a constellation of changes spanning neural development, synaptic function, circadian regulation, and exploratory behaviour, providing a mechanistic framework for interpreting the selective effects on horizontal—but not vertical—exploration described below.

#### 2.3.1. Discrepancy Between the Effects of Bdnf Loss on Vertical and Horizontal Exploration

The present findings reveal a clear dissociation between the vertical and horizontal components of spatial exploration in zebrafish shoals. While vertical positioning followed a similar habituation trajectory in both genotypes, horizontal distribution showed a robust and genotype-specific divergence, with KO shoals displaying an early and transient increase in peripheral occupancy. This axis-specific modulation suggests that vertical and horizontal exploration rely on partially independent behavioural mechanisms and carry different functional significance within the shoaling context. The magnitude of these effects, as indicated by moderate η^2^ values across multiple parameters, suggests that BDNF deficiency induces a systematic shift in spatial behaviour rather than a discrete behavioural separation between genotypes.

Previous studies using the same shoaling paradigm have shown that anxiogenic compounds strongly reduce the exploration of upper-tank regions, whereas anxiolytics increase top-zone occupancy, with minimal effects on horizontal distribution [24]. Such a pharmacological dissociation supports the view that vertical positioning is more tightly linked to anxiety-related processes, while horizontal exploration reflects broader aspects of environmental sampling, social spacing, and group-level decision-making.

In addition, evidence from zebrafish and other shoaling species indicates that vertical positioning is strongly constrained by evolutionarily conserved anti-predatory responses, such as downward displacement following overhead threats, which have been documented in both laboratory and wild fish [27,28,29,30]. The preserved vertical distribution observed in both WT and KO shoals—even in the presence of marked molecular and behavioural alterations—may therefore reflect the robustness of these deeply conserved geotactic and anti-predatory mechanisms, suggesting that *bdnf* loss does not substantially affect these hard-wired defensive programmes.

In addition, both WT and KO shoals displayed two brief increases in group dispersion (around minutes 2 and 8; Figure 4). Although these fluctuations were modest, their timing and recurrence resemble the small-scale adjustments in spacing described in studies of collective behaviour in fish [31,32], where transient changes in cohesion are interpreted as routine vigilance or low-level risk assessment processes rather than full anti-predatory responses. Importantly, the fact that these micro-dispersal events occurred with similar timing and magnitude in both genotypes suggests that *bdnf* loss does not disrupt these basic, species-typical components of collective defensive behaviour.

By contrast, horizontal exploration appears to be more sensitive to modulatory influences such as arousal, social decision-making, and early exploratory drive. The early peripheral peak displayed by KO shoals suggests that *bdnf* loss selectively affects the initial phase of group-level exploration, possibly by altering the balance between risk assessment and environmental sampling.

However, this interpretation should be considered with caution, as it is conceivable that part of the altered horizontal exploration pattern may also reflect broader locomotor or arousal-related alterations previously associated with BDNF deficiency. Previous work in the same model has demonstrated increased locomotion in *bdnf*^−/−^ individuals [18].

In the present study, detailed movement and velocity analyses were not performed due to technical limitations associated with reliably tracking multiple interacting individuals, and therefore these alternative explanations cannot be fully disentangled.

The molecular alterations summarised in Figure 9 provide a plausible mechanistic substrate for this behavioural dissociation. Early transcriptomic dysregulation of neurodevelopmental, synaptic, and circadian pathways, combined with adult proteomic reductions in synaptic and neurotransmission-related proteins, may selectively impact circuits involved in arousal regulation, sensory integration, and spatial decision-making. These systems are likely to contribute more strongly to horizontal exploratory strategies than to vertical geotactic responses, offering a mechanistic explanation for why *bdnf* loss alters the initial dynamics of horizontal exploration while leaving vertical positioning largely preserved. However, direct associations between specific molecular alterations and discrete behavioural outputs cannot be conclusively established in the present study.

Although speculative, this interpretation aligns with evidence in both humans and rodent models showing that *bdnf* and related neurotrophic factors contribute to the fine regulation of intraspecific social behaviour [33,34,35]. Notably, the Val66Met polymorphism in the *bdnf* gene, which regulates activity-dependent BDNF secretion, has been associated with depression and alterations in typical patterns of social interaction [36].

Therefore, the behavioural change observed in KO zebrafish may tentatively be explained by similar effects on social regulation, or by a selective alteration of the mechanisms underlying shoal cohesion and coordination. Finally, since distance from the centroid does not appear to be affected, it is likely that some species-specific shoal characteristics remain robustly unchanged as inherent social constraints [37].

#### 2.3.2. Discrepancies Between Individual and Collective Phenotypes: Integrating Molecular Alterations and Shoaling Behaviour

A second key discrepancy highlighted by the present study concerns the marked difference between the behavioural phenotype of bdnf^−/−^ zebrafish tested individually and that observed at the shoal level. In single-subject paradigms, knockout (KO) adults consistently display increased locomotor activity, reduced anxiety-like behaviour, enhanced boldness, altered stress reactivity, and heightened aggression [18], with these traits robustly replicated across multiple assays. However, when tested in a social context, many of these differences are attenuated: shoal structure, cohesion, and vertical distribution remain largely comparable between KO and WT groups.

This attenuation suggests that social context plays a critical role in modulating the expression of individual behavioural traits. Shoaling provides continuous sensory input, social buffering, and distributed vigilance, all of which can reduce individual variability and constrain behavioural output [38,39,40]. In zebrafish, the presence of conspecifics is known to reduce stress responsiveness and anxiety-like behaviour, leading to more homogeneous group-level dynamics. Such social modulation may override individual predispositions, particularly in strongly social species, resulting in partial phenotypic normalisation at the group level. However, this interpretation remains speculative and is not directly supported by the data presented in the current study. The proposed social buffering effect should therefore be considered as a working hypothesis rather than a conclusion. Experimental approaches such as mixed-genotype shoals would be required to directly assess potential conformity or social buffering mechanisms, but these designs were beyond the scope of the current work due to constraints related to experimental independence and sample size.

Despite this apparent attenuation, the altered horizontal exploration dynamics observed in KO shoals—particularly the early increase in peripheral occupancy—demonstrate that specific components of the individual phenotype persist in the group context. This selective expression indicates that BDNF deficiency does not uniformly affect all behavioural domains but instead modulates specific processes related to spatial exploration, risk assessment, and group-level decision-making.

In this framework, the preservation of shoal cohesion alongside changes in horizontal spatial distribution suggests that the core mechanisms supporting social organisation remain intact, while higher-level exploratory strategies are selectively altered. Integrating these behavioural findings with the molecular alterations depicted in Figure 9 provides a coherent picture: BDNF deficiency induces widespread neurobiological changes that strongly influence individual-level behaviour, while social context constrains and reshapes their expression, revealing only the most robust and socially relevant components of the phenotype.

Some limitations of the present study should be acknowledged. The use of a single shoal size (four individuals per group), although consistent with established three-dimensional shoaling paradigms [24], does not allow an assessment of how different social configurations may influence phenotype expression.

While the number of independent shoals analysed (WT: n = 28; KO: n = 31) provides adequate statistical power and is in line with commonly adopted sample sizes in zebrafish behavioural research [41], future studies incorporating larger and more variable group sizes will be important to further assess the robustness and generalisability of the present findings. The study design was also guided by the principle of reduction (3Rs), aiming to balance statistical sensitivity with ethical considerations by avoiding the use of unnecessary numbers of animals. The use of a shared recirculating housing system further reduces the likelihood that environmental variability contributed substantially to the observed genotype-dependent effects.

Additionally, the shoaling context itself may mask subtle genotype-dependent differences, particularly given that several observed effects were transient and of moderate magnitude.

Furthermore, sex-dependent contributions to the observed phenotype were not specifically investigated. Although a balanced sex ratio was maintained to minimise bias, future studies designed to evaluate males and females separately will be necessary to clarify potential sex-specific effects. Finally, while the 10 min observation window enabled a detailed analysis of early behavioural dynamics, it may not fully capture longer-term adaptations. Extended recording durations and more refined temporal analyses will be important for further characterising the stability and progression of the observed behavioural patterns.

Future studies incorporating heterozygous individuals and varying social group compositions will provide additional insight into gene dosage effects and the interaction between genetic background and social context in shaping collective behaviour.

## 3. Materials and Methods

A total of 236 adult AB zebrafish (*Danio rerio*), 14 months old and with a balanced sex ratio (50:50 male/female), were used in this study. A balanced sex ratio was adopted to minimise potential sex-related biases at the group level, as the present study was not specifically designed to assess sex-dependent behavioural differences.

The sample included 112 wild-type (WT) and 124 *bdnf* knockout (*bdnf*^−/−^) individuals. The *bdnf*^−/−^ mutant line used in this study was the same CRISPR/Cas9-generated line previously established and characterised in earlier publications, where its molecular, physiological, and behavioural phenotypes were extensively documented [18,19]. Zebrafish were raised and maintained according to the European Legislation for the Protection of Animals used for Scientific Purposes (Directive 2010/63/EU) and the Italian animal protection standards (Italian decree 26/2014 updated in 183/2025). The University of Bologna holds license number 270,250/2021 for fish maintenance and breeding. They were maintained in aquaculture housing systems, providing a controlled environment, ensuring a constant temperature of 28 °C and an artificial photoperiod of 13:11 h (light/dark). Fish were housed in 3 L tanks.

All fish used in this study were raised under identical environmental conditions, including tank density, feeding regimen, and light cycle, in order to minimise potential confounding effects on social behaviour. Only wild-type and homozygous *bdnf*^−/−^ individuals were included in the analysis to allow a clear interpretation of the effects of complete *bdnf* loss. Heterozygous fish were not included, as previous work on this line has already demonstrated an intermediate phenotype [18].

Within each genotype, fish were randomly assigned to housing groups and housed in groups of 12 fish per tank.

All tanks were housed within the same recirculating rack system, ensuring that fish of both genotypes were maintained under highly standardised and shared environmental conditions, including identical water supply, temperature, filtration, and light–dark cycle. WT and *bdnf*^−^/^−^ fish were kept in separate tanks to preserve genotype identity and prevent uncontrolled breeding. Within each genotype, fish were randomly distributed across tanks, and experimental shoals were assembled by randomly selecting individuals from different home tanks to minimise potential tank-specific effects.

Fish were fed three times daily (typically 8.30–13:00, 17:30) with commercial dry granular food of different-sized granules (ZM Fish Food & Equipment, Winchester, UK).

### 3.1. Shoaling Behaviour Task

Shoaling behaviour was assessed following the protocol described by Rosa and collaborators [24]. The experimental tank (20 × 15 × 20 cm^3^; width × depth × height) was filled with system water to a depth of 10 cm. All behavioural tests were conducted between 10:00 and 16:00 to minimise circadian variability. Shoals from WT and *bdnf*^−/−^ genotypes were tested in an alternating sequence to reduce potential confounding effects related to testing order. All experimental procedures were conducted under blinded conditions. The operator performing the behavioural testing and the experimenter responsible for video and image analysis were both unaware of fish genotype (see also Section 3.2 for details).

The experimental unit consisted of groups of four fish that were gently netted from their home tank and transferred into a small container and then into the testing tank, where they were released at the centre. Based on the total number of animals, a total of 28 independent shoals (4 fish per shoal) were tested for the WT genotype, and 31 independent shoals for the *bdnf*^−/−^ genotype (Figure 1).

Each shoal was treated as a single independent experimental unit for all statistical analyses. Each fish was tested only once and was not reused across trials, thereby ensuring full independence of observations.

Each trial lasted 10 min and was recorded simultaneously from two orthogonal perspectives using Logitech C922 webcams (Logitech International S.A., Lausanne, Switzerland) positioned 60 cm above (top view) and 60 cm behind (frontal view) the tank. Both cameras were connected to the same computer to ensure synchronised video acquisition.

At the end of each trial, the four fish were transferred to a clean holding tank within the facility. The experimental tank was emptied, rinsed, and refilled with fresh system water before the next trial. No predefined inclusion or exclusion criteria were applied. All animals survived throughout both the maintenance and experimental phases, and consequently no animals, experimental units, or data points were excluded from the final analyses.

### 3.2. Three and Two Dimensional Analysis of Shoaling Behaviour

Video recordings were processed by extracting screenshots every 10 s, yielding 60 pairs of synchronised top view and frontal view images per trial. Image analysis followed the procedure described by Rosa and coworkers [24].

Screenshots were analysed using ImageJ 1.53e software for Windows^TM^. The spatial coordinates of each fish were manually annotated: X and Y from the frontal camera, and X and Z from the top camera. Calibration was performed using the known dimensions of the tank.

For vertical distribution, frontal view images were divided into three equal horizontal zones (bottom, middle, top). For horizontal distribution, top view images were divided into three concentric zones: periphery (<2.5 cm from the tank walls), intermediate area (2.5–5 cm), and centre (>5 cm).

From the coordinate data, we calculated several parameters, including the inter-fish distance in both three and two dimensions, the three-dimensional shoal volume, the shoal area from frontal and top views, each fish’s distance from the centroid, the homogeneity index, and the number of individuals occupying each vertical and horizontal zone.

All calculations were performed using the Excel spreadsheet provided by Rosa et al. [24].

For spatial distribution analyses, the number of fish in each predefined zone was quantified at each sampled time point (i.e., every 10 s snapshot). For each shoal, the reported value corresponds to the mean number of individuals per zone, calculated as the average across all frames of the observation period: mean occupancy = (Σ number of fish in zone at each frame)/total number of frames. Thus, this parameter reflects the average instantaneous distribution of the shoal over time, rather than the cumulative duration of stay in a given area. This approach was preferred over duration-based measures because it provides a time-resolved description of group spatial organisation, capturing how individuals are distributed across zones at each time point. By contrast, duration measures integrate behaviour over time and do not preserve information about the instantaneous configuration of the shoal.

The homogeneity index was used to quantify the spatial uniformity of the shoal. This parameter reflects how evenly individuals are distributed within the available space, with lower values indicating clustering of individuals and higher values indicating a more homogeneous distribution. The index was calculated according to Rosa et al. [24], based on the variance/dispersion of inter-individual distances within the shoal.

All parameters were first computed at the individual level for each frame and then aggregated to obtain a single value per shoal.

Operators responsible for animal housing and husbandry (F.F. and G.B.) were aware of the zebrafish genotype. Conversely, the experimenter conducting the shoaling behaviour experiments (M.T.) and the investigator performing video analyses (A.S.S.G.) were blinded to genotype allocation.

### 3.3. Statistical Analysis

Behavioural parameters were expressed as the mean ± standard error of the mean (S.E.M.). All statistical analyses were conducted using the shoal as the unit of replication, thereby ensuring independence of observations. Genotype effects (WT vs. KO) were assessed using one-way ANOVA for each behavioural parameter.

Temporal dynamics were assessed by calculating the area under the curve (AUC) for each minute of the trial and to evaluate temporal modulation within each genotype, one-way repeated-measures ANOVA was applied across the ten-minute trial (60 data points per trial). For all repeated-measures analyses, the assumption of sphericity was evaluated using Mauchly’s test; when violated, *p* values were corrected using the Greenhouse–Geisser or Huynh–Feldt adjustment based on the ε estimate.

When significant effects were detected, Bonferroni-corrected post hoc tests were applied. Post hoc power analyses were conducted using G*Power 3.1 [42] to estimate the sensitivity of the statistical tests based on sample size and observed effect sizes.

These analyses indicated that, given the number of independent shoals analysed (WT: n = 28; KO: n = 31), the study had sufficient statistical power to detect moderate effect sizes. This is consistent with previous reports indicating that, in zebrafish behavioural studies, n ≈ 12–15 per group is typically sufficient to detect strong effects, while n ≈ 20–25 may be required for smaller effects [41].

In addition, a two-way repeated-measures ANOVA (Genotype × Time) was performed for all parameters to determine whether WT and KO shoals differed in their temporal trajectories. Sphericity was assessed as described above, and Greenhouse–Geisser or Huynh–Feldt corrections were applied when required. When a significant interaction was detected, Bonferroni-corrected post hoc contrasts were used to identify the specific minutes at which KO and WT shoals diverged. Full statistical outcomes are reported in Tables S1–S3.

Statistical outcomes are indicated in Figure 2, Figure 3, Figure 4, Figure 5, Figure 6, Figure 7 and Figure 8 using graphical symbols. Specifically, the asterisk (*) denotes significant Genotype effects (KO vs. WT). The symbols $ and @ indicate significant Time effects relative to minute 1 in KO and WT groups, respectively. Black arrows indicate time points with significant Genotype × Time interactions. Symbols are displayed only when statistical significance is reached.

Statistical analyses were performed using OriginPro 2018 (version 9.5.1.195, OriginLab Corporation, Northampton, MA, USA), RStudio (version 2023.12.1+402; RStudio Team, Boston, MA, USA), and G*Power 3.1 (ver. 3.1). Figures were created using Microsoft PowerPoint 365 (version 2605, Build 16.0.20026.20168, Microsoft Corporation, Redmond, WA, USA).

## 4. Conclusions

The present study demonstrates that the loss of *bdnf* in adult zebrafish produces a selective and domain-specific modulation of shoaling behaviour. First, BDNF deficiency did not alter the structural organisation of the shoal, as inter-fish distance, shoal volume, shoal area, distance to the centroid, and homogeneity index were all comparable between WT and KO groups, although KO shoals lacked the temporal modulation observed in WT fish. Second, vertical spatial distribution was largely preserved, with KO fish showing only a slightly earlier shift toward upper regions of the tank. Third, and most notably, *bdnf* loss induced clear alterations in horizontal spatial distribution, with KO shoals spending more time in peripheral regions and exhibiting a distinctive early peak in peripheral occupancy.

Together, these findings reveal two important discrepancies. The first concerns the differential impact of *bdnf* loss on vertical versus horizontal exploration, suggesting that these two axes of spatial behaviour are regulated by partially independent neural mechanisms and that horizontal exploration is particularly sensitive to BDNF signalling. The second discrepancy concerns the contrast between individual- and shoal-based behavioural outcomes: while KO zebrafish display a robust bold/low-anxiety phenotype when tested individually, these alterations are markedly attenuated in the social context, which may be consistent with a potential social buffering effect. However, this interpretation remains speculative and requires direct experimental validation.

Overall, our results indicate that BDNF is not required for the basic formation or cohesion of the shoal but plays a more nuanced role in shaping the temporal dynamics and spatial strategies of collective exploration. These findings highlight the importance of integrating individual- and group-based assays to fully characterise the behavioural consequences of *bdnf* loss and provide new insights into the neural mechanisms underlying social modulation of behaviour in zebrafish.

## Figures and Tables

**Table 1:** **Summary of statistical outcomes for all behavioural parameters.** The table reports the results of the one-way ANOVA (Genotype effect), one-way repeated-measures ANOVA (Time effect for WT and KO groups), and two-way repeated-measures ANOVA (Genotype × Time interaction). Statistical significance is coded as 1 (significant, *p* ≤ 0.05) and 0 (non-significant, *p* > 0.05). Cells highlighted in green also indicate significant effects. Detailed statistical values for the Genotype effect are provided in Table S1, for the Time effect in Table S2, and for the Genotype × Time interaction in Table S3.

				Effect			
Aspects of Shoal Behaviour		Figure	Parameter	Genotype	Time		Genotype × Time
					WT	KO	
**Shoal structure and cohesion**		1	Inter-fish distance 3D	0	1	0	0
			Inter-fish distance 2D frontal	0	1	0	0
			Inter-fish distance 2D top	0	1	0	0
		2	Shoal volume 3D	0	1	0	0
			Shoal area 2D frontal	0	1	0	0
			Shoal area 2D top	0	1	0	0
		3	Distance to the centroid	0	1	1	0
			Homogeneity index	0	1	1	0
**Spatial positioning**	Vertical	4	Animals in the top area	0	0	1	0
			Animals in the middle area	0	1	1	1
			Animals in the bottom area	0	1	1	0
		5	Centroid in the top area	0	0	0	0
			Centroid in the middle area	0	0	1	0
			Centroid in the bottom area	0	0	1	0
	Horizontal	6	Animals in the periphery area	1	0	1	1
			Animals in the middle area	1	0	1	0
			Animals in the centre area	1	1	1	1
		7	Centroid in the periphery area	1	0	1	0
			Centroid in the middle area	0	0	0	0
			Centroid in the centre area	1	0	0	0

## Data Availability

The datasets generated during the current study are available in the figshare repository (https://doi.org/10.6084/m9.figshare.32089383).

## Supplementary Materials

The following supporting information can be downloaded at: https://www.mdpi.com/article/10.3390/ijms27125464/s1.

## Abbreviations

The following abbreviations are used in this manuscript:

AUC	Area under the curve
BDNF	Brain-derived neurotrophic factor
KO	Knock-out
LDT	Light and dark preference test
MBT	Mirror biting test
NTT	Novel tank diving test
OFT	Open field test
SIT	Social interaction test
SPT	Social preference test
WT	Wild-type
YMT	Y-maze test
