# Supplementary material for: BDNF Deficiency Preserves Shoal Structure but Selectively Modulates Horizontal Exploration in an Adult BDNF−/− Zebrafish Line
Source: Int J Mol Sci. 2026 Jun 17;27(12):5464. doi: 10.3390/ijms27125464 (PMC13299163; doi:10.3390/ijms27125464)
Supplement: Supplementary file 1 [file ijms-27-05464-s001.zip › Figure S1.pdf]

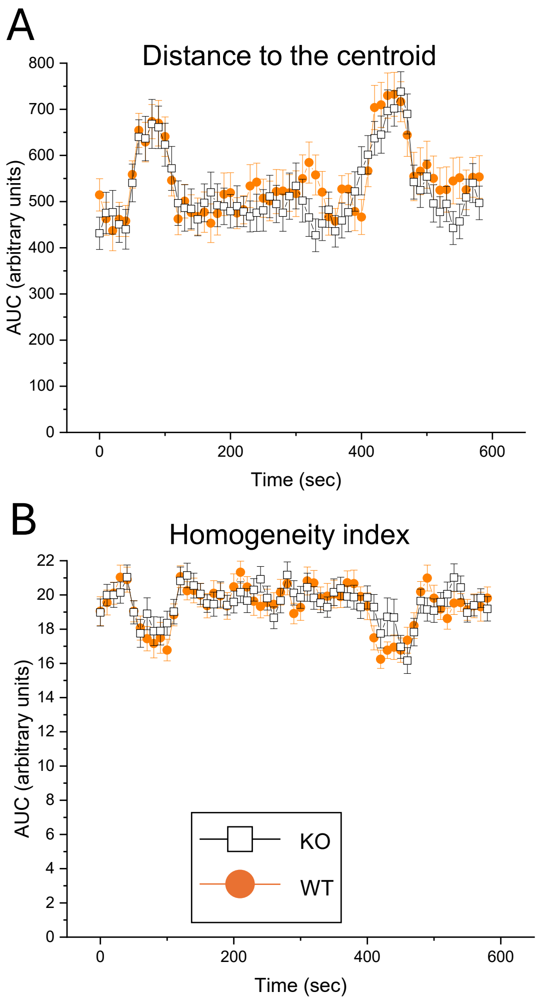

Figure S1. High-resolution temporal dynamics of shoal cohesion parameters. Mean distance to the centroid (A) and homogeneity index (B) for WT and KO shoals plotted at 10-second resolution across the 10-minute observation period. Values are expressed as area under the curve (AUC) calculated for each 10-second interval. Data are presented as mean ± S.E.M. at each sampled time point, with each point representing the average across independent shoals (WT, n = 28; KO, n = 31).
